# Herpetofaunal Community Change in Multiple Habitats after Fifteen Years in a Southwest Florida Preserve, USA

**DOI:** 10.1371/journal.pone.0125845

**Published:** 2015-05-27

**Authors:** John R. Cassani, Dean A. Croshaw, Joseph Bozzo, Brenda Brooks, Edwin M. Everham, David W. Ceilley, Deborah Hanson

**Affiliations:** 1 Florida Gulf Coast University, Department of Marine and Ecological Sciences, Fort Myers, Florida, United States of America; 2 Florida Gulf Coast University, Department of Biological Sciences, Fort Myers, Florida, United States of America; 3 South Florida Water Management District, Estero, Florida, United States of America; 4 Corkscrew Regional Ecosystem Watershed Land & Water Trust, Estero, Florida, United States of America; University of South Dakota, UNITED STATES

## Abstract

Herpetofaunal declines have been documented globally, and southern Florida, USA, is an especially vulnerable region because of high impacts from hydrological perturbations and nonindigenous species. To assess the extent of recent change in herpetofauna community composition, we established a baseline inventory during 1995-97 at a managed preserve in a habitat rich area of southwest Florida, and repeated our sampling methods fifteen years later (2010-11). Nine drift fence arrays were placed in four habitat types: mesic flatwood, mesic hammock, depression marsh, and wet prairie. Trapping occurred daily for one week during 7-8 sampling runs in each period (57 and 49 total sampling days, respectively). Species richness was maintained in mesic hammock habitats but varied in the others. Catch rates of several native species (*Anaxyrus terrestris*, *Lithobates grylio*, *Anolis carolinensis*, *Nerodia fasciata*) declined significantly. Other native species (*Lithobates sphenocephalus*, *Siren lacertian*, and *Notophthalmus viridescens piaropicola*) that were abundant in 1995-97 declined by greater than 50%. Catch rate of only two species (the nonindigenous *Anolis sagrei* and the native *Diadophis punctatus*) increased significantly. Hierarchical cluster analysis indicated similarity within habitat types but significant dissimilarity between sampling periods, confirming shifts in community composition. Analysis of individual species’ contributions to overall similarity across habitats shows a shift from dominance of native species in the 1990s to increased importance of nonindigenous species in 2010-11. Although natural population fluctuations may have influenced differences between the two sampling periods, our results suggest considerable recent change in the structure and composition of this southwest Florida herpetofaunal community. The causes are unknown, but hydrological shifts and ecological impacts of nonindigenous species may have contributed.

## Introduction

Global herpetofauna declines, especially among amphibians, have been well documented at various spatial scales and in diverse habitat types [1–8]. Current amphibian declines and extinctions greatly exceed background rates, and as many as one third of amphibian species have been affected severely [9–10]. This trend has prompted the recognition of an amphibian decline crisis in the context of maintaining community biodiversity [4]. Declines of reptile populations around the world have also been documented, with increased calls for monitoring and vigilance [2, 8, 10].

Adams et al. [11] concluded that overall pond occupancy of amphibians in the United States declined 3.7% annually between 2002 and 2011, and red-listed species, as determined by the International Union for Conservation of Nature, declined by an average of 11.6% annually during the same period. Gardner et al. [12] and Dodd and Smith [13] suggested that habitat change is the primary cause of population decline of reptiles and amphibians worldwide, although additional factors may contribute. For example, environmental contamination, *UV-B* irradiation, disease, introduced species, exploitation, and climate change are all likely influential [4]. Additionally, complex synergistic effects among multiple causes have the potential to impact amphibian populations [14].

In Florida, USA, herpetofaunal population declines have been documented and largely attributed to habitat change or loss and the influence of nonindigenous species (e.g., [15–23]. Statewide loss of historic wetlands was estimated at 44% [24], and in southwest Florida, wetlands comprise a major habitat for herpetofauna. Estimates of historic wetland loss in the region approach 50% resulting from the direct impacts of land use conversion and indirectly from altered hydrology [25]. Such profound and recent landscape changes have the potential to impact amphibian and reptile communities severely. Increased inventory and monitoring efforts can provide information that will allow informed management to counter such threats.

Synoptic studies of herpetofauna and their associated habitats have occurred in northwest Florida where species assemblages and habitat types closely represent those of the southeastern USA region [26–27]. However, similar studies in peninsular and south Florida have been lacking since 2000. The rarity of such studies is a major concern because Florida, particularly south Florida, has experienced significant development and landscape alterations over the past half century, especially in coastal areas and concentrated agricultural regions south of Lake Okeechobee [15]. Moreover, animal communities in the region are experiencing strong invasion pressure from nonindigenous species, a force likely to exact major changes on native amphibian and reptile populations [3, 28]. Although considerable research in the region has aimed at examining the spread and potential impacts of these invaders [29–34] and anecdotal evidence of species replacement is common, we know of few long-term studies that document recent shifts in amphibian and reptile community composition in peninsular Florida. Long-term data sets are critical resources for conservation biologists, and more of these efforts are needed. Indeed, monitoring initiatives spanning at least 7–10 years are required for a reasonable chance to detect population trends that can be separated from natural fluctuations [35–37].

One strategy is to identify sites that were subject to intense sampling in the past and target them for resampling followed by comparisons of faunal changes (e.g., [26, 38]). We adopted this approach in southwest Florida, USA, a region that may have experienced significant recent change due to landscape alteration and increased prevalence of nonindigenous species. The objectives of our study were: 1) to establish a current herpetofauna inventory in a large managed preserve of diverse habitats, and 2) to examine whether these communities have changed after 15 years by comparing species richness and composition at four habitat types using the same methodology in 1995–97 and 2010–11. We further examined changes in relative abundance, as determined by counts, of nonindigenous species and speculate on their potential influences on native species. Our data provide an assessment of recent community change as well as a baseline for long-term monitoring of the impacts of an ever-increasing nonindigenous fauna in south Florida.

## Methods

### Study area

Drift fence arrays were established within the Corkscrew Regional Ecosystem Watershed (CREW) management area, a 24,000 ha preserve straddling Lee and Collier Counties, Florida, USA. CREW is managed by the South Florida Water Management District in cooperation with the CREW Land and Water Trust, as well as the Florida Fish and Wildlife Conservation Commission. One of the management goals of CREW is wildlife conservation, and our study addresses this element of the management plan. Field sampling of all nine sites on this public land resource was approved by and conducted in cooperation with the CREW Trust, South Florida Water Management District and the Florida Fish and Wildlife Conservation Commission. These state and regional agencies represent the public stewardship and regulation of the preserve. None of the sampled biota was harvested nor was any endangered species captured, precluding the need for a “listed species” permit. Habitat features of CREW are dominated by a 2,000 ha sawgrass marsh at the headwaters of the watershed. The remainder is composed of a diverse mosaic of hammock, flatwoods, swamp, marsh and prairie habitats. The large size, diversity and mostly undisturbed history of CREW made it an ideal site to study herpetofauna in remnant habitats that most closely resembled the pre-developed state of the region. Documenting such changes in relatively high value habitats should help prioritize conservation strategies, not only for CREW but also for similar remnants of south Florida. A total of nine arrays were sited among four primary habitat types (Table 1) based on descriptions of vegetation communities by the Florida Natural Areas Inventory [39]. Priorities for array locations were reasonable access, contrasting degrees of inundation and homogeneity of habitat type. Annual rainfall in this region of Florida is high and concentrated in the rainy season months of June-September. This study was approved by the Florida Gulf Coast University Institutional Animal Care and Use Committee (permit 0708–09).

**Table 1:** CREW Drift fence array labels, locations (WGS 84) and habitat types.

Array	(N) Latitude	(W) Longitude	Habitat Type
C1	26.47932	81.54906	Mesic Flatwoods
C2	26.47408	81.54265	Mesic Flatwoods
C3	26.47901	81.54572	Mesic Flatwoods
C4	26.47408	81.54265	Mesic Hammock
C5	26.48276	81.53802	Mesic Hammock
C6	26.45423	81.55603	Mesic Hammock
C7	26.45260	81.55277	Depression Marsh
C8	26.45394	81.54918	Depression Marsh
C9	26.45551	81.55325	Wet Prairie

Chosen sites were further inventoried prior to study initiation in 1995 to assess plant species composition and abundance, height of understory and ground cover, canopy occlusion, and presence of nonindigenous flora. Nonindigenous plants were maintained at minimal coverage with selective herbicides subsequent to the 1995–97 sampling period. Habitats within CREW, particularly pine flatwoods and marsh communities, are selectively burned every four to seven years to mimic natural fire regimes and maintain existing vegetation communities.

**Fig 1:**
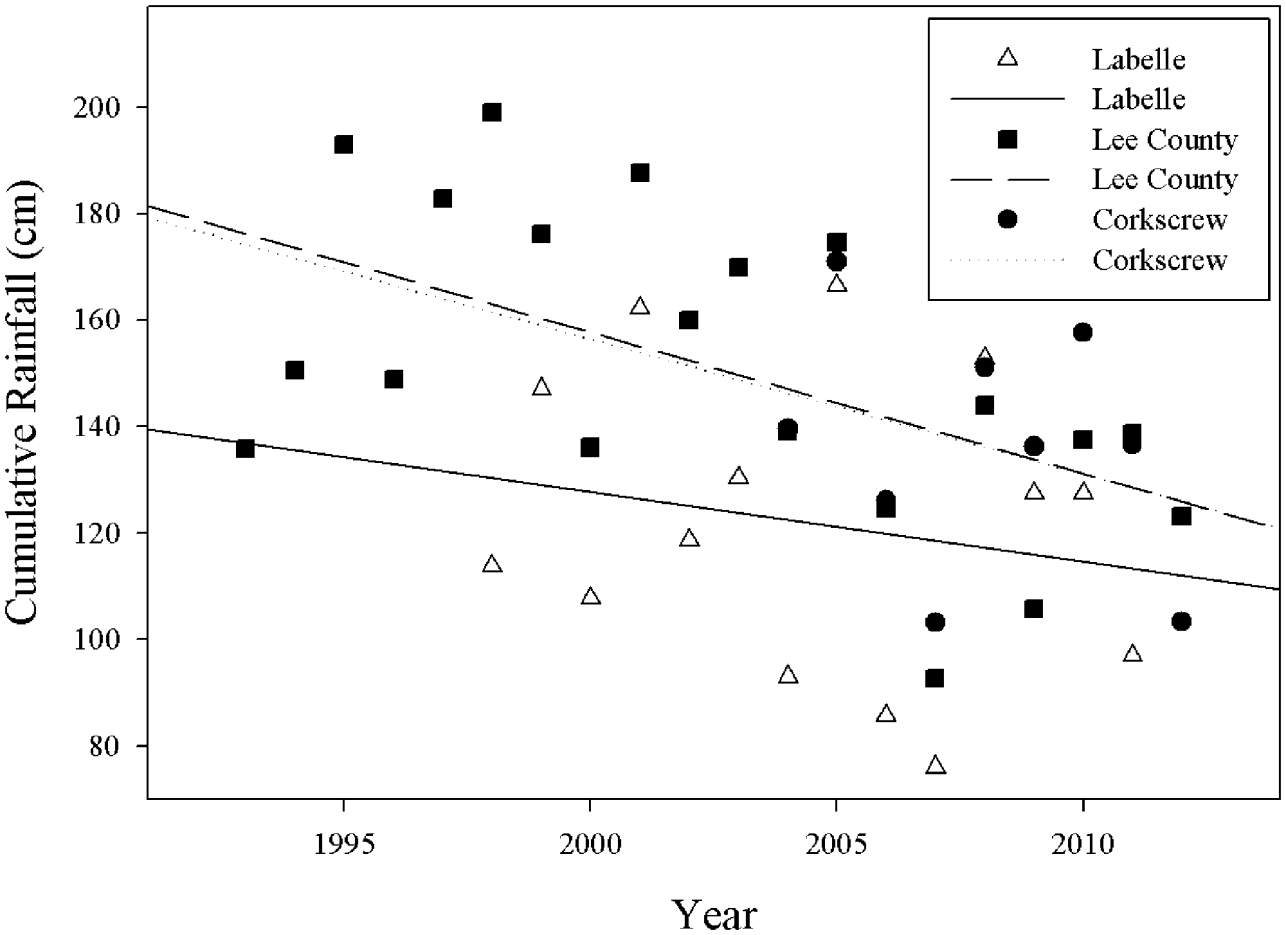
Cumulative yearly rainfall and best-fit regression lines from three locations proximal to the CREW preserve. All three locations represent aggregated data from multiple sampling sites.

The 1995–97 sampling period occurred during a period of relatively abundant rainfall (Fig 1, Lee County), but it was followed by two periods of severe drought. November 1999 through May 2001 was, at the time, the driest recorded sequence of dry-wet-dry seasons in south Florida [40]. Later, 2006–07 were the driest back-to-back calendar years Florida has experienced since 1932 [41]. Clearly, the latter sampling period occurred during a time of considerably less rainfall. Wetland hydroperiods and other hydrological parameters important to reptiles and amphibians in CREW were likely affected. However, substantial change to the vegetation communities associated with the nine array sites through 2010 was not apparent. The author (JB) who established the sites for sampling in 1995 and was involved in subsequent preserve management determined that very little structural change had occurred to the habitats and that the same dominant plant species were present.

### Data collection

Drift fence arrays were composed of buried silt fencing supported by wooden stakes and configured in a cross orientation (four arms) with each arm approximately 7.6 m in length. Two funnel traps (mouth 7–8 cm diameter) composed of aluminum screen were located at the end of each array arm. Traps were shaded with vegetation, and a wet cloth was placed inside to prevent desiccation of trapped animals.

Traps were open for seven or eight consecutive days per sampling run and monitored either each day or every other day. Sampling runs from 1995–97 occurred during the months of February, March, June, August, September, November and December. In 2010–11 sampling runs occurred during February, April, June, July, August, September and December. Traps were set for a total of 57 days in 1995–97 and 49 days in 2010–11. Animals were released unmarked near the capture site but several meters from the array to minimize incidental recaptures.

We gathered precipitation data from several weather stations in the region to compare rainfall during the two sampling rounds and the interim period.

### Data analysis

Species collected during the two sampling periods (1995–97 and 2010–11) were evaluated in the context of changes in community composition and relative abundance from count data. Margalef and Shannon diversity indices were used to describe herpetofauna diversity at each array and for each sampling period, which allowed documentation of changes over time as well as comparisons among habitats. The rarefaction option in the software Primer, version 6 [42], was used to account for differences in total captures between the two sampling periods.

We used two statistical approaches to explore whether individual amphibian and reptile species showed evidence of decline or expansion, McNemar’s G Test [43] and paired t-tests on catch rate data. McNemar’s test uses presence-absence data and is based on the null hypothesis that the number of arrays switching from present to absent is equal to the number of arrays switching from absent to present if population size did not change. Here and throughout the paper, the word “absent” indicates undetected by our drift fence sampling methods. Catch rate was expressed as the number of individuals of each species trapped at the nine array sites per 7- or 8-day sampling run in both of the two sampling rounds.

Multivariate analyses were conducted using Primer. We considered species count data to be a reasonable proxy for “abundance” and use that term throughout. Abundance data were normalized based on sampling effort and fourth-root transformed to down-weigh the importance of extremely abundant species [44]. A Bray-Curtis similarity matrix [45–46] was then used as a basis for comparison of the herpetofaunal communities among habitats and between time periods. Hierarchical agglomerative cluster analysis (using group average linking) based on Bray-Curtis similarities was employed to examine natural groupings among habitats and sampling periods. The groupings produced in the cluster diagram were further evaluated for statistically significant evidence (p < 0.05) of genuine clusters in sample assemblages using a series of similarity profile (SIMPROF) random permutation tests. The Similarity Percentage Test (SIMPER) was used to identify the contributions of individual species in forming the Bray-Curtis similarity matrix as well as the similarity and dissimilarity within and among sampling sites and sampling periods. Non-metric multidimensional scaling (MDS) was also employed. This statistical tool can be based on Bray-Curtis similarity matrices and has been recommended for use in examining communities structured by environmental gradients [43–44].

## Results

Thirty-four and 33 species were captured during the 1995–97 and 2010–11 sampling periods, respectively (Table 2; S1 Dataset). Twenty-seven species were common to both sampling periods. A relatively abundant species in 1995–97 (sixth most abundant), *Notophthalmus viridescens piaropicola*, was not found during 2010–11. Species that were rare or relatively uncommon in 1995–97 and absent in 2010–11 included *Anolis carolinensis*, *Ophisaurus ventralis*, *Chelydra serpentina osceola*, *Pseudacris nigrita*, *Sistrurus miliarius barbouri* and *Terrapene carolina bauri* (Table 2). Relatively common species (12 most abundant) in 1995–97 that decreased by more than 50% in 2010–11 were *Lithobates grylio*, *Lithobates sphenocephalus* and *Siren lacertina*. Species that were rare or absent in 1995–97 but relatively abundant in 2010–11 were *Anaxyrus quercicus* and *Anolis sagrei*.

**Table 2:** Species abundance for the two sampling periods and results of McNemar’s test for population decline or expansion and paired t-tests of catch rate data (p-values).

	1995–97			2010–11				
Species	Total individuals	Ind. per day	Arrays	Total individuals	Ind. per day	Arrays	McNemar’sTest	Catch Rate Test
**Anura**								
*Acris gryllus*	47	0.82	C1–9	40	0.82	C1–7, C9	1.00	0.90
*Anaxyrus quercicus*	5	0.09	C1, C3	36	0.73	C1–4, C6	0.25	0.07
*Anaxyrus terrestris*	8	0.14	C1–2, C4, C6, C8	4	0.08	C1, C4, C6	0.50	0.05
*Eleutherodactylus planirostris*	271	4.75	C1–6, C8–9	334	6.82	C1–9	1.00	0.33
*Gastrophryne carolinensis*	86	1.51	C1–9	181	3.69	C1–9	1.00	0.32
*Hyla cinerea*	10	0.18	C1–2, C4, C9	1	0.02	C6	0.38	0.14
*Hyla femoralis*	0	0	-	2	0.04	C2–3	0.50	0.17
*Hyla squirella*	0	0	-	3	0.06	C1, C4–5	0.25	0.08
*Lithobates grylio*	107	1.88	C1–9	13	0.27	C1–3, C6–8	0.25	0.002
*Lithobates sphenocephalus*	428	7.51	C1–9	93	1.90	C1–9	1.00	0.08
*Pseudacris nigrita*	3	0.05	C1–2	0	0	-	0.50	0.04
*Pseudacris ocularis*	11	0.19	C1–3, C8	3	0.06	C2, C8	1.00	0.21
**Caudata**								
*Amphiuma means*	2	0.04	C8	5	0.10	C7	1.00	0.57
*Notophthalmus viridescens piaropicola*	61	1.07	C7–9	0	0	-	0.25	0.21
*Siren lacertian*	144	2.53	C1, C7–9	11	0.22	C7–8	0.50	0.14
**Lacertilia**								
*Anolis carolinensis*	12	0.21	C1–3, C6–9	0	0	-	0.02	0.002
*Anolis sagrei*	2	0.04	C3, C5	85	1.73	C1–9	0.02	0.001
*Ophisaurus compressus*	1	0.02	C2	1	0.02	C7	1.00	0.93
*Ophisaurus ventralis*	1	0.02	C2	0	0	-	1.00	0.35
*Plestiodon inexpectatus*	26	0.46	C1–6	13	0.27	C1–6	1.00	0.31
*Scincella lateralis*	9	0.16	C1, C4–6	2	0.04	C1–2	0.63	0.31
**Serpentes**								
*Cemophora coccinea*	6	0.11	C2–3, C8	3	0.06	C3–4, C7	0.63	0.59
*Coluber constrictor priapus*	47	0.82	C1–4, C6–9	25	0.51	C1–6, C8–9	1.00	0.06
*Diadophis punctatuspunctatus*	0	0	-	17	0.35	C1–9	0.004	0.0003
*Lampropeltis elapsoides*	0	0	-	2	0.04	C3–4	0.50	0.17
*Nerodia fasciata pictiventris*	23	0.40	C1, C2, C4–5, C7–9	7	0.14	C7–8	0.06	0.04
*Nerodia floridana*	4	0.07	C7, C9	1	0.02	C7	1.00	0.19
*Pantherophis alleghaniensis*	8	0.14	C2–3, C8	2	0.04	C5–6	1.00	0.35
*Pantherophis guttatus*	1	0.02	C2	4	0.08	C7, C9	1.00	0.37
*Regina alleni*	0	0	-	1	0.02	C7	1.00	0.35
*Storeria victa*	2	0.04	C7	1	0.02	C5	1.00	0.73
*Sistrurus miliarius barbouri*	1	0.02	C7	0	0	-	1.00	0.35
*Seminatrix pygaea cyclas*	1	0.02	C9	3	0.06	C8–9	1.00	0.17
*Thamnophis sauritus sackenii*	29	0.51	C2–9	29	0.59	C1, C3–6, C8–9	1.00	0.73
*Thamnophis sirtalis sirtalis*	13	0.23	C1, C6–8	2	0.04	C5, C8	1.00	0.28
**Testudines**								
*Chelydra serpentina*	2	0.04	C7, C9	0	0	-	0.50	0.17
*Kinosternon baurii*	18	0.32	C1, C4, C5, C7–9	9	0.18	C2–3, C5, C7, C9	1.00	0.49
*Kinosternon steindachneri*	1	0.02	C8	1	0.02	C1	1.00	0.93
*Sternotherus odoratus*	1	0.02	C7	3	0.06	C5, C8	1.00	0.41
*Terrapene carolina bauri*	1	0.02	C9	0	0	-	1.00	0.35

McNemar’s test with presence-absence data yielded just one species that showed significant decrease (*A*. *carolinensis*, Table 2), two species that showed significant increase (*A*. *sagrei* and *Diadophis punctatus*), and one that showed marginally significant decrease (*Nerodia fasciata*). Catch rate with count data yielded four species with significant decreases (*A*. *carolinensis*, p = 0.002; *Anaxyrus terrestris*, p = 0.05; *L*. *grylio*, p = 0.002; *N*. *fasciata*, p = 0.04) and two species with marginally significant decreases (*L*. *sphenocephalus*, p = 0.08; *Coluber constrictor*, p = 0.06). Two species showed significant increases (*A*. *sagrei*, p = 0.001; *D*. *punctatus*, p = 0.0003) and one species had a marginally significant increase (*A*. *quercicus*, p = 0.07). Overall, the catch rate decreased for 17 of the 27 species common to both study periods (63%).

SIMPER analysis for 1995–97 indicated that anurans represented the top five species contributing to similarity among habitats (Table 3). They had the greatest average abundance and greatest habitat similarity contributions. *Lithobates sphenocephalus* and *L*. *grylio* were the top two species in both measures (Table 3). The SIMPER analysis of the 2010–11 period resulted in a shift in species average abundance and importance ranking and a smaller increase in similarity between habitat types compared to the earlier 1995–97 period. *Lithobates sphenocephalus* remained relatively common, ranking second in contribution to habitat similarity, but *L*. *grylio* dropped out of the list of important species. The nonindigenous *Eleutherodactylus planirostris* had the greatest average abundance and contribution to habitat similarity during 2010–11. *Anolis sagrei*, an additional nonindigenous species not among the most abundant species in 1995–97, ranked third during 2010–11. In the context of dissimilarity, species that were relatively common in 1995–97 but rare in 2010–11 (or vice versa) ranked highest (Table 3). *Anolis sagrei* and *L*. *grylio* are relatively highly ranked in their contributions to a shift in herpetofauna community structure among habitats between the two time periods.

**Table 3:** SIMPER results comparing mean abundance of herpetofauna collected between 1995–97 and 2010–11 and species contributions to the dissimilarity among habitat types between the two sampling periods (total average dissimilarity = 48.44%).

Species	Mean Abundance		Contribution %	Cumulative %
	1995–97	2010–11	dissimilarity between periods	
*Anolis sagrei* ^2^	0.22	1.68	7.03	7.03
*Diadophis punctatus punctatus* ^2^	0	1.15	5.64	12.67
*Lithobates grylio* ^1^	1.79	0.78	5.06	17.73
*Eleutherodactylus planirostris* ^1^ ^,^ ^2^	1.77	2.17	4.84	22.57
*Siren lacertina*	0.97	0.32	4.78	27.35
*Anolis carolinensis*	0.88	0	4.26	31.62
*Nerodia fasciata pictiventris*	0.96	0.29	4.14	35.76
*Anaxyrus quercicus*	0.27	0.85	4.06	39.81
*Lithobates sphenocephalus* ^1^ ^,^ ^2^	2.4	1.76	3.6	43.41
*Plestiodon inexpectatus*	0.92	0.77	3.48	46.89
*Kinosternon baurii*	0.82	0.62	3.23	50.13
*Notophthalmus viridescenspiaropicola*	0.66	0	3.03	53.16
*Pseudacris ocularis*	0.56	0.24	2.87	56.03
*Anaxyrus terrestris*	0.61	0.35	2.87	58.9
*Thamnophis sauritus sackenii*	1.19	1.02	2.86	61.76
*Thamnophis sirtalis sirtalis*	0.55	0.22	2.76	64.53
*Scincella lateralis*	0.51	0.22	2.65	67.18
*Hyla cinerea*	0.54	0.11	2.59	69.77
*Coluber constrictor priapus*	1.34	1.15	2.57	72.34
*Gastrophryne carolinensis* ^1^ ^,^ ^2^	1.68	1.8	2.48	74.82
*Cemophora coccinea cocccinea*	0.38	0.33	2.38	77.21
*Pantherophis alleghaniensis*	0.41	0.22	2.34	79.54
*Acris gryllus* ^1^	1.44	1.26	2.15	81.7
*Hyla squirella*	0	0.33	1.6	83.3
*Pantherophis guttatus*	0.11	0.26	1.58	84.88
*Seminatrix pygaea cyclas*	0.11	0.24	1.54	86.42
*Nerodia floridana*	0.26	0.11	1.5	87.93
*Sternotherus odoratus*	0.11	0.24	1.48	89.41
*Amphiuma means*	0.13	0.17	1.28	90.7

Species accounting for similarity between habitat types within each sampling period are indicated by superscripts.

^1^. Five species accounting for 56.9% cumulative similarity between habitats during the first sampling period

^2^. Five species accounting for 61.8% cumulative similarity between habitats during the second sampling period

The SIMPER analysis indicated a mean among-habitat similarity in 1995–97 of 59.38% with more than 50% of that similarity driven by five species (Table 3): *L*. *sphenocephalus* (15.10%), *L*. *grylio* (12.03%), *Gastrophryne carolinensis* (11.09%), *Acris gryllus* (9.63%) and the nonindigenous *E*. *planirostris* (9.05%). The mean similarity among sample sites (all habitats) in the second sampling period (2010–11) was 61.16% with more than 50% of the similarity resulting from four species: *E*. *planirostris* (14.52%), *L*. *sphenocephalus* (13.66%), *A*. *sagrei* (12.44%) and *G*. *carolinensis* (12.31%). The analysis also indicated an average dissimilarity between 1995–97 and 2010–11 of 48.44%. To account for more than 50% of that dissimilarity required twelve species (Table 3). Two of those 12 species are nonindigenous and increased in abundance during the study: *A*. *sagrei* and *E*. *planirostris*. Eight native species decreased in abundance between the two sampling periods. Only two native species, *A*. *quercicus* (0.27 to 0.85) and *D*. *punctatus* (0.00 to 1.15), increased in abundance from 1995–97 to 2010–11.

Species richness (Margalef’s) increased at all mesic hammock sites (C4–6) but decreased at two of the three mesic flatwood sites (C1 and C2, Table 4). Richness decreased at one of the two depression marsh sites and at the wet prairie site. Shannon diversity, which incorporates relative abundance and may be influenced by the inherent sampling bias of drift fences, increased at all mesic hammock sites (C4–6) and one mesic flatwood site (C3) but declined at all other sites.

**Table 4:** Changes in univariate measures of the herpetofauna community (species richness, Margalef Richness, and Shannon diversity index) from 1995–97 (1995 in table) and 2010–11 (2010 in table), separated by array site.

Array	No. Species		Margalef Richness		Shannon Diversity	
	1995	2010	1995	2010	1995	2010
C1	18	15	5.28	4.72	2.86	2.67
C2	19	14	5.62	4.32	2.90	2.60
C3	14	15	4.36	4.63	2.59	2.67
C4	13	14	3.78	4.33	2.49	2.58
C5	11	15	3.53	4.54	2.31	2.64
C6	12	14	3.77	4.38	2.43	2.56
C7	17	16	4.97	5.01	2.78	2.75
C8	19	14	5.58	4.52	2.93	2.61
C9	17	11	4.95	3.56	2.77	2.33

**Fig 2:**
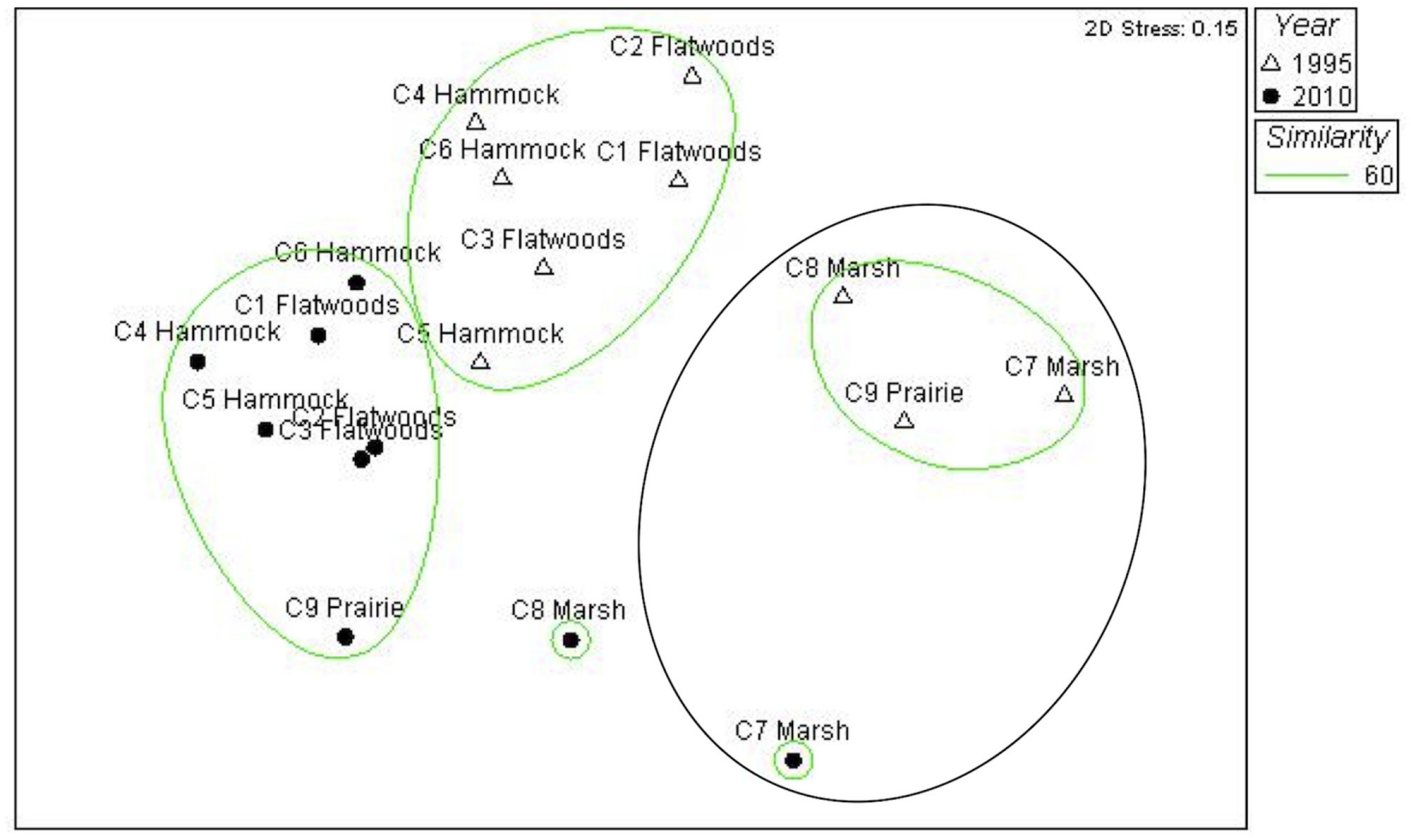
Non-metric multi-dimensional scaling ordination based on Bray-Curtis similarity. Samples are labeled by array number, habitat, and year. Circles identify groupings that hierarchical cluster analysis indicated to be significantly different from one another at the 95% confidence level.

With the exception of marsh habitat C7, all herpetofauna communities represented by habitat types were significantly different (p < 0.05) between the two time periods (Fig 2). The MDS ordination indicated a similar separation of communities by sampling period and by elevation.

## Discussion

Our study documents statistically significant changes in amphibian and reptile populations at this study site in southwest Florida, USA. Nonindigenous species increased in importance after a relatively short period between sampling rounds (15 years) and several native species declined precipitously. Our results suggest that herpetofaunal communities are changing rather quickly in southwest Florida. Despite these shifts in community structure, overall biodiversity as defined by species richness appears to have been partially maintained at CREW after the 15-years between sampling. Species richness was variable across habitats but not to a great degree. Small increases were shown at all hammock sites (C4, C5, C6), one marsh site (C7), and one flatwoods site (C3) whereas the other habitats showed declines (flatwoods C1 and C2, depression marsh C8 and wet prairie C9). More importantly, catch rate of most native species declined. It remains unclear whether these differences reflect true regional population declines, natural fluctuations, or environmental influences. Because our sampling represented two periods in time rather than a continual 15-year study, these conclusions could be susceptible to yearly variation in amphibian and reptile populations. Nevertheless, we trapped over multiple seasons and years in each period and our results thus suggest the possibility that biological diversity could be compromised eventually at this and other highly invaded communities in the region.

Because our traps only captured animals small enough to physically enter the funnel opening (7–8 cm diameter), the documented number of amphibian and reptile species likely is conservative. Our methods excluded especially large snakes and most adult turtles. Arboreal species such as treefrogs are also underrepresented [47]. Drift fence funnel traps do not capture all species at the same rate but effectively capture some individuals of most species and can be used to compare relative abundance of species among study areas [48].

The CREW management area is a regional remnant of mostly undisturbed habitat. Different habitat types associated in spatial proximity provide microclimates, microhabitats and dispersal corridors that contribute to increased herpetofauna abundance and species richness [47–52]. Habitat heterogeneity at CREW is relatively high due to a mix of upland habitats and wetlands characterized predominantly by depression marshes. Habitats are maintained with prescribed burns to mimic natural fire regimes. Our study confirms that the CREW management plan implementation, along with minimal human disturbance, has resulted in one of the few remaining areas of relatively high biodiversity in the southwest Florida region.

However, changes in herpetofaunal community composition (as indicated by temporal dissimilarity in our analysis) can occur while species richness remains relatively stable. Species contributing most to such changes were those that were new, absent or experienced fluctuation in abundance, potentially from environmental influence, competitive interaction, or a combination of both factors.

Forys and Allen [53] used cross-scale resiliency theory to quantify how loss of native vertebrate species of south Florida and invasion by nonnatives may alter functional group richness within and across scales. They predicted that functional group richness will not change significantly within scales despite large changes in species composition, nor will there be any significant loss of overall functional redundancy across scales. However, the types of functions performed could change and may have profound effects on the entire landscape of south Florida. Though we examined fewer functional groups than Forys and Allen [53], the compositional changes we documented among herpetofauna may have similar implications for ecological function and reorganization.

The latter sampling period, 2010–11, was preceded by severe droughts in 2001–02 and 2006–07. Drought can change regional hydrology by causing hydroperiods to shorten, thus compromising normal wetland function. Amphibian species requiring relatively long hydroperiods to complete development or to sustain life history requirements are typically impacted the most [53–54]. Not surprisingly, species contributing the most to dissimilarity between the two periods were those requiring relatively long hydroperiods such as *L*. *grylio*, *Siren lacertina* and *Notophthalmus viridescens piaropicola*. Furthermore, because recruitment of metamorphic juvenile amphibians is known to vary drastically with rainfall [55], it is possible that decreases in abundance for species such as *A*. *terrestris*, *L*. *sphenocephalus*, and *L*. *grylio* reflect lower metamorph production rather than declines in adult populations.

Abundance of nonindigenous species, *E*. *planirostris* and *A*. *sagrei*, increased during the latter sampling period of 2010–11. The multivariate analysis shows an increase of importance of these two nonindigenous species in accounting for the similarity within habitats, and a corresponding importance in the dissimilarly between the two sampling periods. Overall the multivariate community analysis indicates a trend toward increasing importance of nonindigenous species.

Interspecific competition between *A*. *sagrei* and *A*. *carolinensis* may be at least partly responsible for the apparent disappearance of *A*. *carolinensis* at CREW [29]. Our study suggests that more work should be focused on native anole conservation and population ecology. We did not employ arboreal sampling and thus could not have detected *A*. *carolinensis* populations in high microhabitats. The hope remains that these lizards persist in the face of competition and predation from *A*. *sagrei* by shifting habitat use. *Eleutherodactylus planirostris* was relatively abundant during both sampling rounds, but clearly increased in importance between samplings. Although we know of no studies showing negative impacts of *E*. *planirostris* on native fauna, their populations do appear to be expanding. These increases may be partly responsible for the appearance of *D*. *punctatus* during the 2010–11 period, as they have been reported to feed on *E*. *planirostris* in other areas of Florida [28]. *Diadophis punctatus* also may have benefitted from the increase in *A*. *sagrei* whose eggs serve as a food source [56].

Species that were present in 1995–97 but appeared to decline greatly in the latter study period may offer priorities for species conservation management or protection. *Pseudacris nigrita*, which was rare during 1995–97 and absent in 2010–11, has been shown to be extremely rare or extirpated from the region by frog call surveys [37]. *Notophthalmus viridescens piaropicola* is a species more recently in decline possibly in response to drought. Although our sampling design did not detect them in the latter period, one individual was collected at the C8 array in 2011 as part of ancillary sampling [57]. Species with known occurrence in this region but not collected during either time period may also warrant closer scrutiny. *Heterodon platirhinos* was identified by Wilson and Porras [15] as having undergone a population reduction in south Florida. In addition, we are aware of only two *H*. *platirhinos* observations from cursory road kill surveys and other unpublished ancillary faunal surveys in southwest Florida over the past 15 years. Similarly, *H*. *simus* has become rare in Florida and elsewhere [58] and is being considered as a state “listed” species by the Florida Fish and Wildlife Conservation Commission.

*Lampropeltis getula*, *Opheodrys aestivus* and *Aspidoscelis sexlineata* were all notably absent in our data set. The first is another snake in serious decline or locally extirpated in Florida [59]. *Opheodrys aestivus* was relatively common during a multi-month road kill survey in 1993 (JRC, unpublished data) but may also be in significant decline. To our knowledge only two other specimens have been seen since 1993, one by a local resource manager at the Caloosahatchee Creeks Preserve, Lee County, Florida on September 16, 2010 (C. Olson, personal communication) and another at C-44 in southeast Florida. *Aspidocelis sexlineata* has become a rare sighting over recent years as well. Additional studies in south Florida are needed to verify declines of these species and to examine potential causes while recovery efforts are still feasible.

Our study is not the first to resample the same sites as historical work; however, it is among the few that used the same methods and some of the same workers. Such research has been rare recently in peninsular and south Florida. Comparing data sets is especially difficult when precise details of methodology are unknown as is often the case for historical ecological inventories. Thus, many similar studies can offer only limited inferences about community change over decades and often focus on presence/absence and biological diversity. The differences we documented do not suffer from these constraints and allow robust statistical tests of ecological change. Thus, our work serves as a notable case study documenting shifts in a highly invaded continental community. We hope that further research will continue to document the response of south Florida’s native herpetofauna to pressure from environmental shifts and introduced species.

## Supporting Information

S1 Dataset Herpetofauna captures at Corkscrew Regional Ecosystem Watershed. Species are listed by common name and an abbreviation of the scientific name. Habitats are ordered by their designations (Table 1). Years refer to the sampling period: 1995: 1995–96; 2010: 2010–11.
(XLSX)
